# Efficient visible light photocatalysis of benzene, toluene, ethylbenzene and xylene (BTEX) in aqueous solutions using supported zinc oxide nanorods

**DOI:** 10.1371/journal.pone.0189276

**Published:** 2017-12-20

**Authors:** Jamal Al-Sabahi, Tanujjal Bora, Mohammed Al-Abri, Joydeep Dutta

**Affiliations:** 1 Department of Petroleum and Chemical Engineering, College of Engineering, Sultan Qaboos University, Al-Khoudh, Oman; 2 Chair in Nanotechnology for Water Desalination, Water Research Center, Sultan Qaboos University, Al-Khoudh, Oman; 3 Center of Excellence in Nanotechnology, Asian Institute of Technology, Klong Luang, Pathumthani, Thailand; 4 Functional Materials, Department of Applied Physics, School of Engineering Sciences, KTH Royal Institute of Technology, Kista, Stockholm, Sweden; Institute of Materials Science, GERMANY

## Abstract

Benzene, toluene, ethylbenzene and xylenes (BTEX) are some of the common environmental pollutants originating mainly from oil and gas industries, which are toxic to human as well as other living organisms in the ecosystem. Here we investigate photocatalytic degradation of BTEX under visible light irradiation using supported zinc oxide (ZnO) nanorods grown on glass substrates using a microwave assisted hydrothermal method. ZnO nanorods were characterized by electron microscopy, X-ray diffraction (XRD), specific surface area, UV/visible absorption and photoluminescence spectroscopy. Visible light photocatalytic degradation products of BTEX are studied for individual components using gas chromatograph/mass spectrometer (GC/MS). ZnO nanorods with significant amount of electronic defect states, due to the fast crystallization of the nanorods under microwave irradiation, exhibited efficient degradation of BTEX under visible light, degrading more than 80% of the individual BTEX components in 180 minutes. Effect of initial concentration of BTEX as individual components is also probed and the photocatalytic activity of the ZnO nanorods in different conditions is explored. Formation of intermediate byproducts such as phenol, benzyl alcohol, benzaldehyde and benzoic acid were confirmed by our HPLC analysis which could be due to the photocatalytic degradation of BTEX. Carbon dioxide was evaluated and showed an increasing pattern over time indicating the mineralization process confirming the conversion of toxic organic compounds into benign products.

## Introduction

Petroleum industries are contributing largely in the global economy as well as in the development of oil-producing countries. Millions of barrels of crude oil are produced worldwide every day, which contains potentially toxic chemicals used in drilling, as well as natural contaminants ejected from the oil wells, including total dissolved solids (e.g., salts, barium, strontium), organic compounds (mainly aliphatic and aromatic hydrocarbons) and normally occurring radioactive material (NORM), such as Radium 226 [1–4]. Aromatic hydrocarbons in crude oil is composed of trace amounts of polycyclic aromatic hydrocarbons (PAHs) that are relatively less soluble in water and high concentrations of monoaromatic hydrocarbons such as benzene, toluene, ethylbenzene and xylenes (BTEX) that are grouped as volatile organic compounds (VOCs), some of which are relatively soluble in water. Both groups contribute to environmental contamination when released into the environment finding their way to water, soil and air [5]. Benzene, toluene, ethylbenzene, and xylene (i.e., BTEX), though present in low percentages in crude oil are of high interest as they have been associated with adverse human health effects [6]. Produced water from oilfields and refinery wastewater usually contain some amounts of monoaromatic hydrocarbons as well [7–9]. Lined holding ponds that are constructed at well sites for temporary storage of the produced water are potential sources of surface spills and leaks of BTEX-containing liquids and also have the potential to leak into aquifers due to structural failure of casings and/or stray gas migrations [10]. Benzene can threaten human health and is mutagenic and carcinogenic [5]. It has a high vapor pressure that can rapidly contaminate the surrounding air, which can then lead to water and soil contamination via rainfall. Other BTEX compounds are derivatives of benzene which also show toxic effects. Benzene (5 ppb), toluene (1000 ppb), ethylbenzene (700 ppb) and xylene (10,000 ppb) are the permissible limits of drinking water maximum contaminant level (MCL) [11].

Different treatment technologies are in use for BTEX removal, such as biological treatment, chemical adsorption and advanced oxidation processes (AOPs) [12–16]. Among these, AOPs are a cluster of processes that produce high reactive hydroxyl radicals (OH^•^) leading to the destruction of harmful organic contaminants present in test matrix [17–19]. Photocatalysis is one of the popular AOPs for converting organic pollutants into harmless products, like CO_2_, H_2_O and mineral acids [20,21]. Furthermore, it is cost-effective, safe, non-selective and compatible to treat a broad range of organic contaminants [22–24]. Semiconductor photocatalysis lead to the mineralization of organic pollutants when it is irradiated with ultraviolet (UV) or visible light [25]. Carbon nanotubes (CNTs) also have been reported as an active nanomaterial for photocatalytic degradation of toxic organic contaminants and for sensing of heavy metals such as chromium and aluminum (III) ions in water [26–28]. Zinc oxide (ZnO), titanium dioxide (TiO_2_) and tin dioxide (SnO_2_) are the most widely used wide bandgap semiconductor photocatalysts [29,30]. The use of UV lamps is expensive and not practical for large area applications. Sunlight in retrospect, can be a viable cost-effective solution for large scale use of photocatalysis for the degradation of organic matters, but it contains only about 5% UV and 45% visible light [31]. Since ZnO is a wide bandgap semiconductor, modification of the material is necessary to make it visible light active in order to harvest major part of the solar spectra. Many studies are available in the literature offering different strategies to render ZnO materials visible light active. For example, coupling of ZnO with plasmonic metals [32,33], metal and non-metal doping [34,35], composites with another semiconductors [36–39], and self-doping by inducing crystal defects [40–44].

Nanotechnological application using zinc oxide nanomaterial got much respective attention and particularly in medical investigation and its applications, photocatalysis and material science. Zinc oxide is applied as anticancer agents [45,46]. Zinc oxide is widely used for photocatalytic degradation of toxic contaminants in water [47–50]. It is also used as antibacterial activity and antifouling [51,52], gas sensing [49,53] and for flame transport approach [48,54].

Photocatalytic degradation of BTEX, whether individually or as a group, in gaseous samples using UV light have been intensively studied, while very little research has been done to investigate BTEX degradation in aqueous solutions and that too with visible light [55–57]. Toluene was reported to be more degradable than benzene when visible light photocatalysis is used for aqueous solution [57]. Although BTEX is volatile, its presence is always detected in the wastewater produced typically in oil and gas industries. In the present study, we therefore probe the photocatalytic degradation of BTEX in aqueous medium using visible light active defect engineered ZnO nanorods [58]. ZnO nanorods were hydrothermally grown on microscopic glass substrates and used as supported photocatalyst. Degradation of BTEX in aqueous solution as individual components was then investigated and discussed. Intermediate formation of byproducts was also evaluated using a mixed BTEX solution and evolution of carbon dioxide as a result of complete mineralization of BTEX was probed by gas chromatograph equipped with thermal conductivity detector (TCD).

## Materials and methods

### Synthesis of zinc oxide nanorods

Microwave assisted hydrothermal (MAH) process for the synthesis of ZnO nanorods is discussed in our previous reports [41,58,59]. Microscope glass substrates were successively cleaned with soap water, ethanol, acetone and finally with deionized (DI) water in an ultrasonic water bath for 15 minutes respectively. The clean glass substrates were then placed on a hotplate at 350°C and a ZnO seed layer was deposited on them by direct spraying of 10 mM zinc acetate dihydrate [Zn(CH_3_COO)_2_.2H_2_O; Merck] aqueous solution. The ZnO seeded glass substrates were then dipped in an aqueous solution comprising of 20 mM zinc nitrate hexahydrate [Zn(NO_3_)_2_·6H_2_O; Sigma] and 20 mM hexamethylenetetramine (HMTA; Merck). The reaction vessel was then heated in a commercial microwave oven (Samsung model# ME731K, serial # J68C7WFC5015084Y) operated at 180 W (growth solution temperature: 90°C) for 45 minutes and then allowed to cool down naturally for 15 minutes [58]. The growth solution was then replenished with a fresh solution and the heating process was continued for another 4 similar cycles [60]. After the 5th cycle of microwave treatment, the glass substrates were removed, rinsed with DI water and dried in an oven at 90°C. The as prepared ZnO nanorod coated glass substrates were then annealed at 350°C in air for 1 hour in order to improve the visible light photocatalytic (VLP) activity of the nanorods. The annealing temperature was fixed based on our previous results where we have shown that the VLP activity of the ZnO nanorods can be improved through temperature induced defect migration from the bulk to the surface of ZnO nanorods [41].

### ZnO nanorod characterization

Scanning electron microscopy (SEM, JEOL JSM-7600F) operated at 20 kV was used to investigate the morphology of the synthesized ZnO nanorods. X-ray diffraction technique (XRD, Rigaku miniflex 600) with Cu Kα radiation (λ = 0.154 nm) was used to study the crystal structure of the nanorods. XRD pattern was recorded in the 2θ range from 20° to 80° in 0.02°/s steps. The specific surface area was determined using nuclear magnetic resonance (NMR) technology from Xigo Nanotools. Field emission transmission electron microscope (TEM, JEOL JEM-2100F) was used for the morphological structure, crystal nature and the lattice fringe spacing. A double beam UV-visible spectrometer (Perkin Elmer Lambda 25) was used to measure the steady state optical absorption spectrum of the ZnO nanorods. Photoluminescence (PL) spectra were collected at room temperature using a fluorescence spectrometer (Perkin Elmer LS 55). ZnO nanorods were excited with 325 nm monochromatic wavelength in order to obtain PL spectra at ambient conditions.

### Photocatalytic degradation tests for BTEX

**Fig 1:**
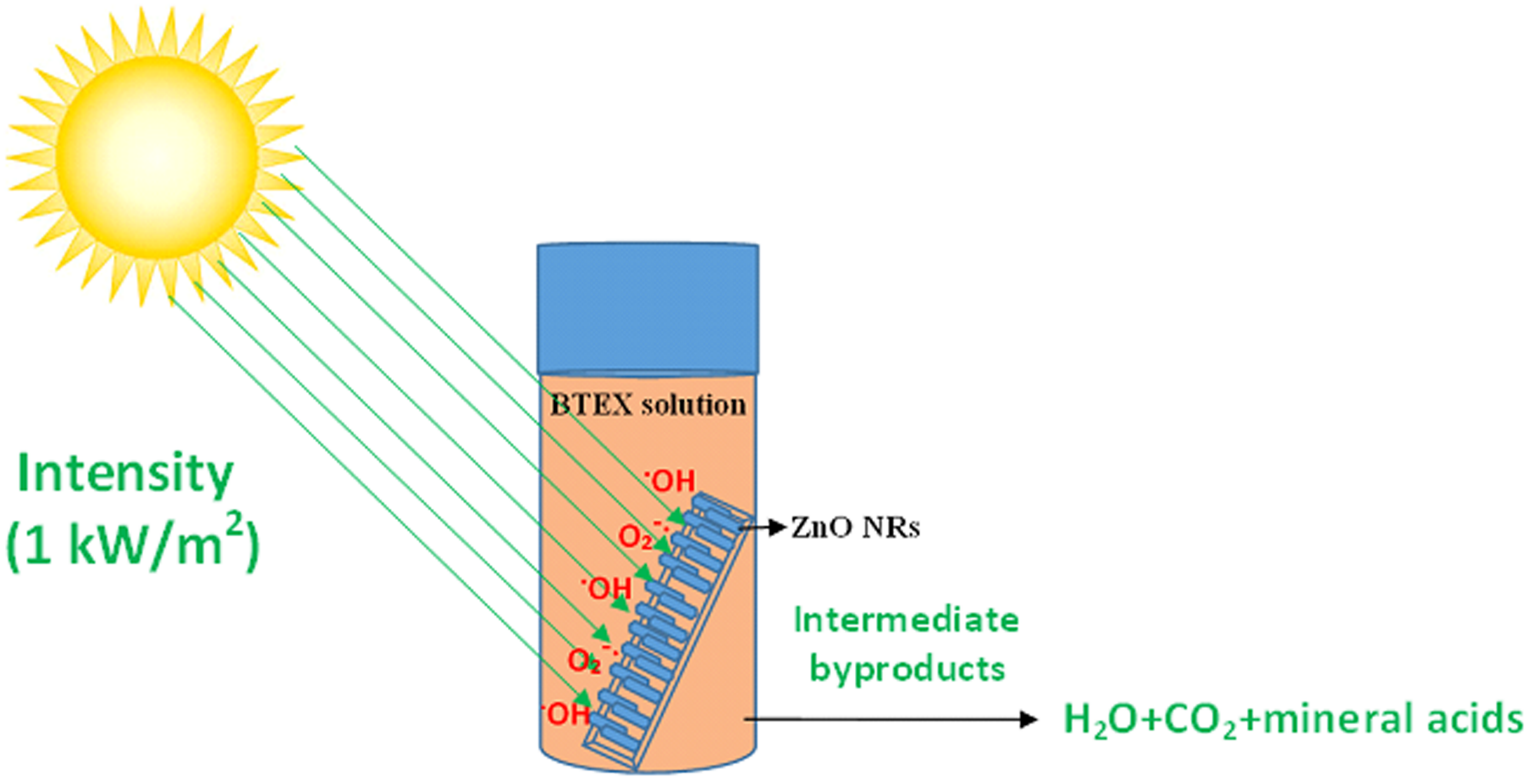
Schematic representation of a photocatalytic process using zinc oxide nanorods for degrading the monoaromatic hydrocarbons.

An aqueous benzene solution (10 ppm) was prepared and a 25-ml sealed glass bottle was filled with the benzene solution following which the bottle was sealed with a septum cap. Prior to the sealing, a ZnO nanorod coated glass slide (dimensions of 4 cm x 1.25 cm) was placed vertically in the bottle. The sealed bottle was then stored in dark at room temperature for 2 hours in order to obtain equilibrium between the benzene molecules adsorption and desorption at the ZnO surface. After 2 hours, the bottle was illuminated with artificial solar irradiation (incident power: 1 kW/m^2^) using a Sciencetech solar simulator (SS1.6 kW). Another similar sealed glass bottle filled with 10 ppm benzene aqueous solution in the absence of any photocatalyst was used under similar conditions as control sample. Photocatalytic degradation of benzene was continued up to 3 hours and at regular intervals 300 μl of benzene aliquots were collected with a syringe to analyze the reduction in benzene concentration due to the photocatalytic reactions. Similar steps were applied for photocatalytic degradation of toluene, ethylbenzene and xylene under visible light irradiation. The initial concentration of the test contaminants was then varied from 10 to 100 ppm by keeping all the other parameters same in order to investigate the effect of initial concentration of the contaminants on the kinetics of the photocatalytic reactions. Degradation kinetics was studied by analyzing the VOC contents using gas chromatograph-mass spectrometer (GC/MS) technique. A mixture of BTEX solution (25 ppm) was also used to test for intermediate byproduct formation and carbon dioxide evolution during the photocatalytic degradation of BTEX. The experimental details were illustrated (Fig 1).

### Analytical methods

Photocatalytic reduction of BTEX with time was monitored by gas chromatograph equipped with mass spectrometer (GC/MS, Shimadzu model# QP2010 Ultra) along with an autosampler (Shimadzu model# AOC-20i) injecting 1 μl sample into the GC. A polar capillary column (Rtx-Wax, 30 m length, 0.25 mm I.D., 0.25 μm film thicknesses, Restek) was used for BTEX analysis. High purity helium was used as the carrier gas (99.9999%) with a flow rate of 1 ml/min. The injector, MS interface and ion source temperatures were 250, 250 and 220°C, respectively. The initial oven temperature was set at 60°C and increased to 150°C by slowly heating at 6°C/min (15 minutes run time). The mass-spectroscopic (MS) fragments were scanned in 35–300 amu. Spectral library (NIST14 database) was used to identify the compounds of interest. All results were then plotted as C_t_/C_o_ against time t, where C_t_ represents concentration of each monoaromatic hydrocarbon at a given time interval and C_o_ represents their starting concentration.

The intermediate products formed during photocatalysis were evaluated by high performance liquid chromatography (HPLC) equipped with an autosampler (SIL-30A, Shimadzu, Tokyo, Japan) and an ODS hypersil column (particle size: 3 μm, internal diameter: 4.6 mm, length: 80 mm) from Hewlett Packard, Santa Clara, CA, USA. The mobile phase was prepared by mixing water and methanol in a ratio 50:50 and was degassed before use. The flow rate of the mobile phase was 0.6 mL/min. 20 μl of samples were injected into the system each time and the sample was scanned from 200 to 400 nm to probe the intermediates using a photodiode array detector (SPD-M20A) from Shimadzu, Japan.

For detecting the carbon dioxide evolved as a result of complete mineralization of BTEX, gas chromatograph with thermal conductivity detector (Agilent 6890N) was used. 250 μl of the headspace gas, using gas-tight syringe, was injected every 30 minutes into a HP-PLOT Q column (HP-PLOT Q, 30 m length, 0.53 mm internal diameter, 40 μm film thicknesses, J&W Scientific). The set flow rate of helium (99.9995%) was 4 ml/min in a split mode (split ratio 10:1). The temperatures of the injection port and the detector were kept at 200°C and 210°C, respectively. The initial oven temperature was set at 50°C and slowly increased to 80°C (holding for 3 minutes) at a rate of 20°C/min. The total run time was 4.5 minutes.

## Results and discussion

**Fig 2:**
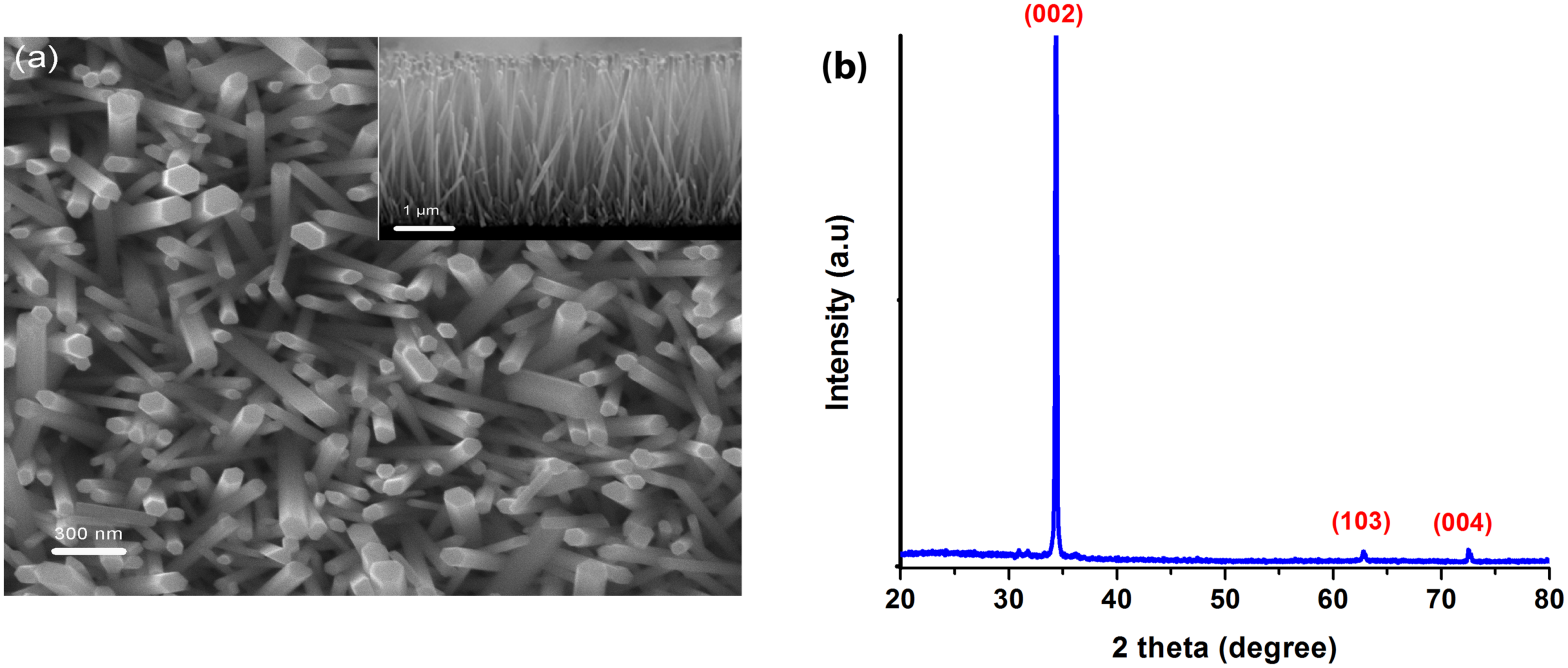
(a) Top and cross-sectional (inset) SEM micrographs and (b) XRD pattern of ZnO nanorods. (a) SEM micrographs of ZnO nanorods and (b) XRD pattern of the microwave assisted hydrothermally grown ZnO nanorods on glass substrate. Samples were annealed at 350°C for 1 h after the hydrothermal growth.

The morphology of the as prepared ZnO nanorods was studied using scanning electron microscope (SEM) and vertically aligned ZnO nanorods growing from the glass substrates could be observed, as shown in Fig 2(a). The ZnO nanorods have an average length of about 4.5 μm as shown in the inset, with the characteristic hexagonal shape of ZnO nanorods being evident with an average diameter of about 100 nm. Fig 2(b) shows the XRD pattern of the ZnO nanorods where the diffraction peaks conform to the hexagonal crystal structure of ZnO nanorods as confirmed with the Joint Committee on Powder Diffraction Standards (JCPDS card# 01-070-8070). The preferential orientation of the ZnO nanorods along the (002) crystal plane is indicated by the strongest XRD peak at θ = 34.35°C representing the (002) plane of the wurtzite crystal structure [61,62]. The specific surface area of the synthesized zinc oxide nanorods was determined by using Xigo nanotools system that works based on NMR technique and found to be 4.2 ± 0.5 m^2^/g.

**Fig 3:**
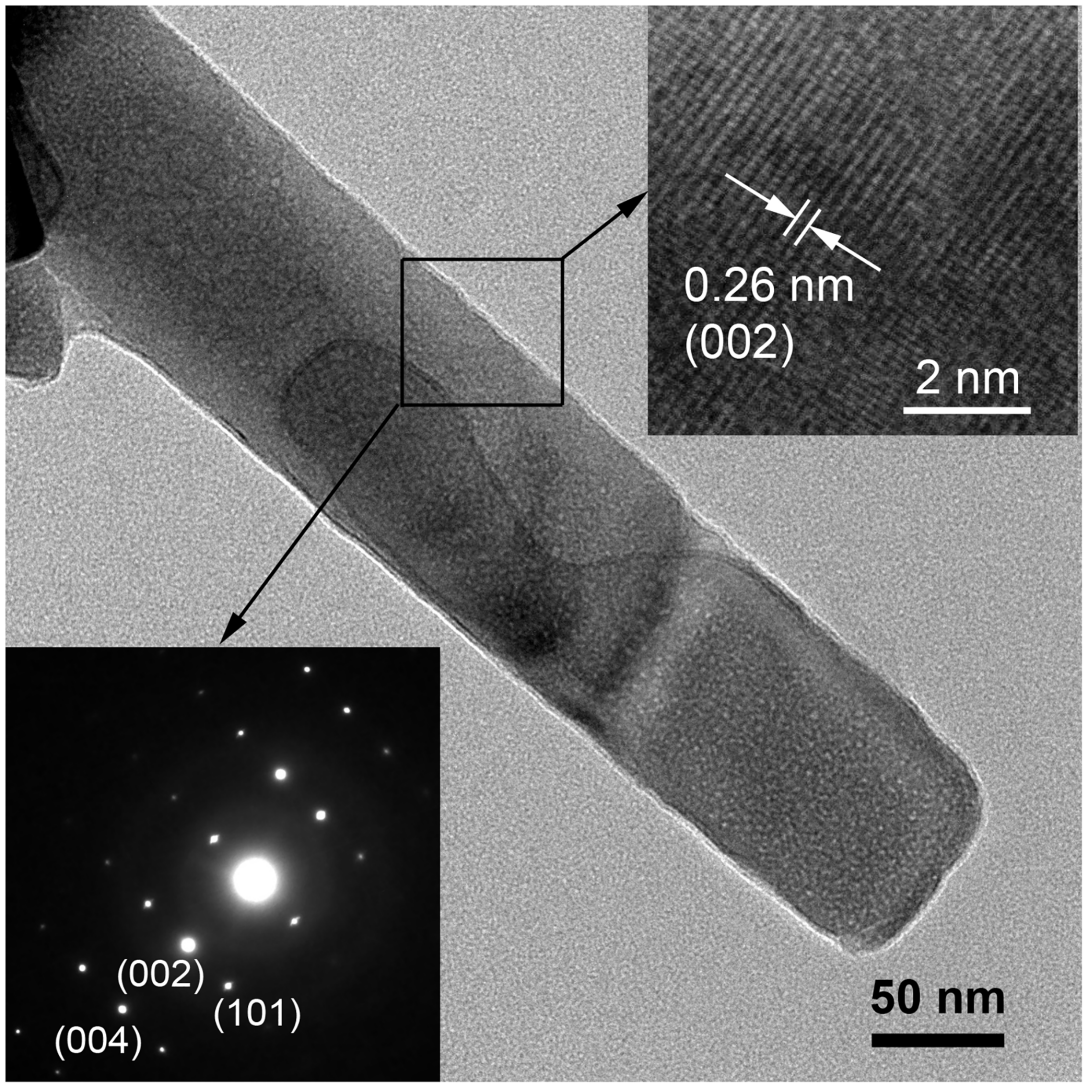
TEM micrograph of a synthesized single nanorod. The top inset representing the lattice fringes in the ZnO nanorods and the bottom inset is for the high resolution TEM showing the SAED pattern.

Fig 3 reveals that the low resolution TEM micrograph confirming the structural formation of ZnO nanorods where the diameter is about 100 nm. The clear lattice fringe spacing (d-spacing) is 0.26 nm confirming the hexagonal wurtzite structure and the presence of the dominant (002) plane [63] was detected. The selected area electron diffraction (SAED) pattern is assuring a single crystal formation of ZnO nanorods [64], synthesized by MAH process, as was observed using high resolution TEM (HRTEM).

**Fig 4:**
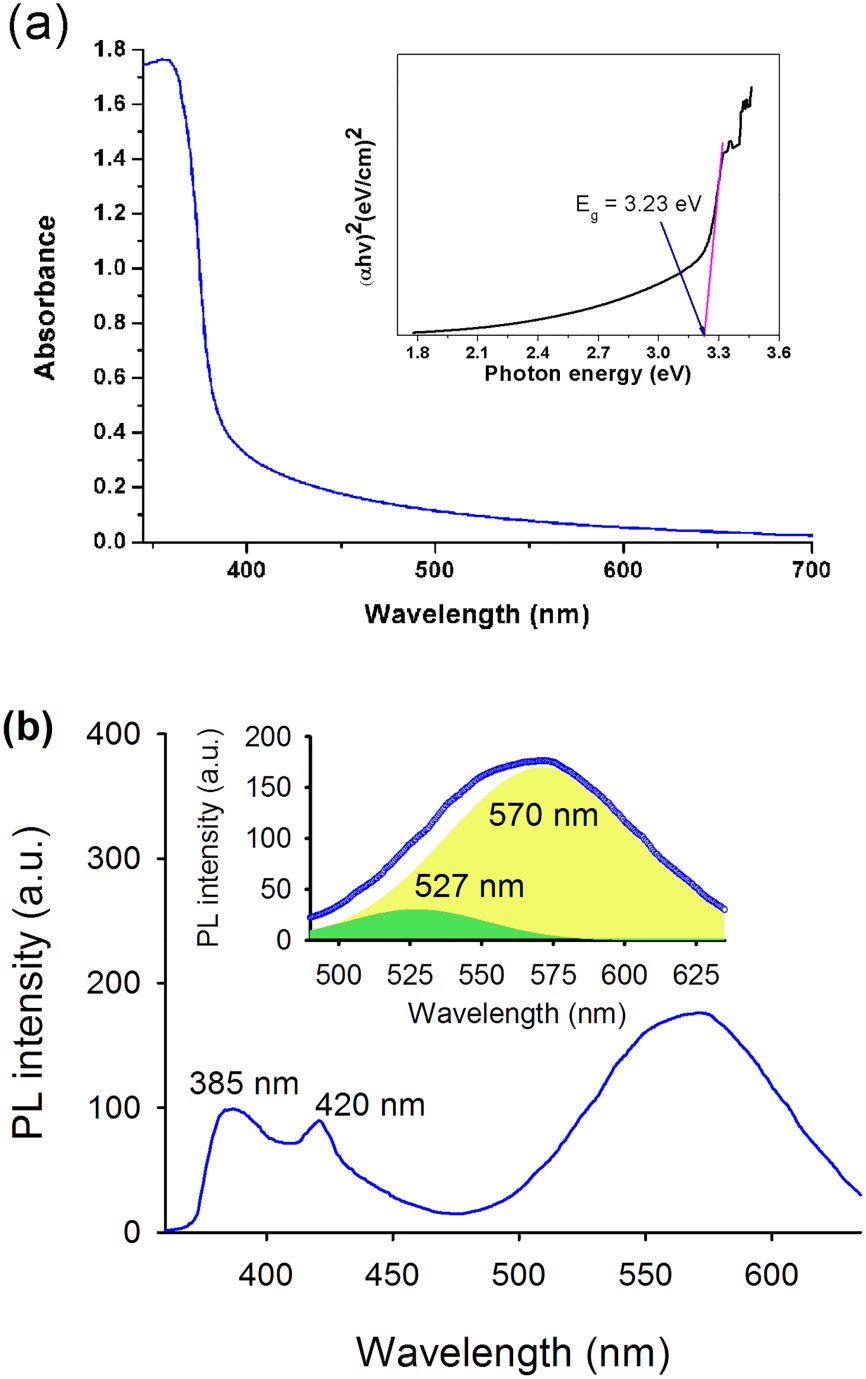
(a) Typical optical absorption spectrum and (b) room temperature photoluminescence (PL) spectrum of ZnO nanorods. Inset in Fig 4a representing the tauc plot and inset in Fig 4b is showing the ZnO surface defect mediated PL bands (excitation: 325 nm) deconvoluted into two Gaussian components centered at 527 nm and 570 nm, respectively.

Fig 4(a) shows the UV/Visible optical absorption spectra of the zinc oxide nanorod coated substrates. The nanorods exhibit strong absorption above 385 nm and show an extension of the absorption edge into the visible region. The bandgap (Eg) of ZnO was found to be around 3.23 eV obtained by using tauc plot [65] as shown in Fig 4 (inset). Similar values of bandgap was also reported previously in literature [50,66]. The visible light absorption by ZnO nanostructures have been attributed due to the presence of native point defects in the crystal lattice of ZnO which reduce the energy required for exciton pair generation upon photo-excitation [67,68]. The visible light activity of ZnO with respect to the concentration of defect states is also reported by Tang et al. [69] where they have demonstrated reduction in the optical bandgap of ZnO with increasing oxygen vacancy states.

In order to investigate the presence of defect states in the synthesized and annealed ZnO nanorods, we measured the photoluminescence (PL) spectrum of the nanorods. Fig 4(b) shows the room temperature steady state PL spectrum of ZnO nanorods measured with 325 nm monochromatic light. The PL spectrum exhibit three major peaks, where the absorption around 385 nm that can be attributed to the direct recombination of excited electrons from the conduction band (CB) to the valence band (VB) of the ZnO nanorods. The origin of the violet emission (at 420 nm) is attributed to the deep-level zinc interstitial (Zn_i_) defects that are slightly below the CB of ZnO [70]. This emission occurs due to the capture of excited electrons from the CB of ZnO by Zn_i_ defect states through non-radiative transition followed by a radiative recombination of the electrons from the defect states to the VB. The broad emission in the range from 490 nm to 635 nm, which is found to compose of two Gaussian components centered at 527 nm and 570 nm respectively is mainly due to the surface situated oxygen vacancy states, where singly charged oxygen vacancies (V_O_^+^) are responsible for the 527 nm emission and the 570 nm emission originates from the oxygen vacancy states with doubly charged states (V_O_^++^) [67,71].

**Fig 5:**
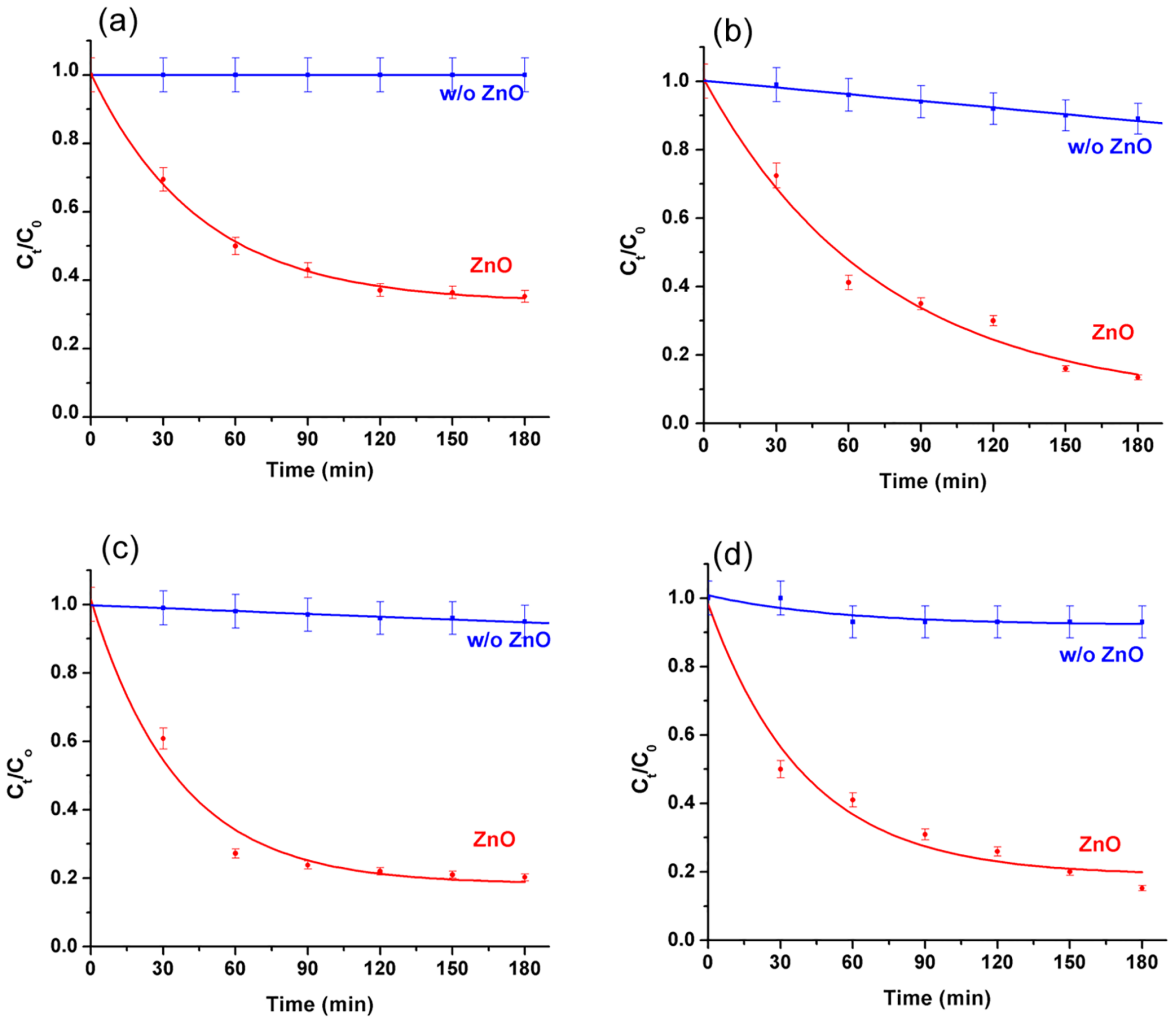
Reduction in the concentrations of (a) benzene, (b) toluene, (c) ethylbenzene and (d) xylene. Reduction in the concentration of (a) benzene, (b) toluene, (c) ethylbenzene and (d) xylene under simulated solar light irradiation (incident power: 1 kW/m^2^) in the presence and absence of ZnO nanorods. The starting concentration of all the monoaromatic hydrocarbons was 25 ppm.

The VLP activity of the ZnO nanorods was then studied for BTEX removal in aqueous medium and detected by GC/MS (Supporting information S1 Fig). Fig 5 shows the degradation profile of the individual monoaromatic hydrocarbons (initial concentration: 25 ppm) in the presence and absence of ZnO nanorods under simulated solar irradiation. For 25 ppm benzene, in the absence of ZnO nanorods, negligible amount of benzene was found to degrade during 3 hours of visible light irradiation carried out in these experiments. On the contrary, in the presence of ZnO nanorods, 50% degradation of benzene was observed in about 1 hour exhibiting almost 1.75 times higher degradation. Other three monoaromatic hydrocarbons (toluene, ethylbenzene and xylene) also showed similar degradation trends in the presence of ZnO nanorods. Based on the percentage removal of each monoaromatic hydrocarbons upon 3 hours of photocatalytic treatment, we found that both toluene and xylene were degraded almost equally by the ZnO nanorods exhibiting ~90% reduction in their concentrations within 3 hours, followed by ethylbenzene (~80%) and finally benzene (~65%). The instrumental detection limits of benzene, toluene, ethylbenzene and xylene by using GC/MS were tested and found 10, 5, 10 and 10 ppb (μg/L) respectively.

The photocatalytic degradation of BTEX as a function of initial concentration of the test contaminants in the presence of ZnO nanorods was then investigated by using Langmuir-Hinshelwood (L-H) kinetics model. The L-H model has been previously used for heterogeneous photocatalysis to explain the bimolecular reaction of two surface adsorbed species [72–74]. The model is mathematically given as:

$$\frac{1}{k_{obs}}=\frac{1}{k_{c}K_{LH}}+\frac{A_{o}}{k_{c}}$$ (1)

where A_o_ is the initial concentration of the test contaminant (in mg.L^−1^), k_obs_ is the apparent pseudo-first-order rate constant, k_c_ is the rate constant of surface reaction (in mg.L^−1^.min^−1^) and K_LH_ is the Langmuir–Hinshelwood adsorption equilibrium constant (in L.mg^−1^).

**Fig 6:**
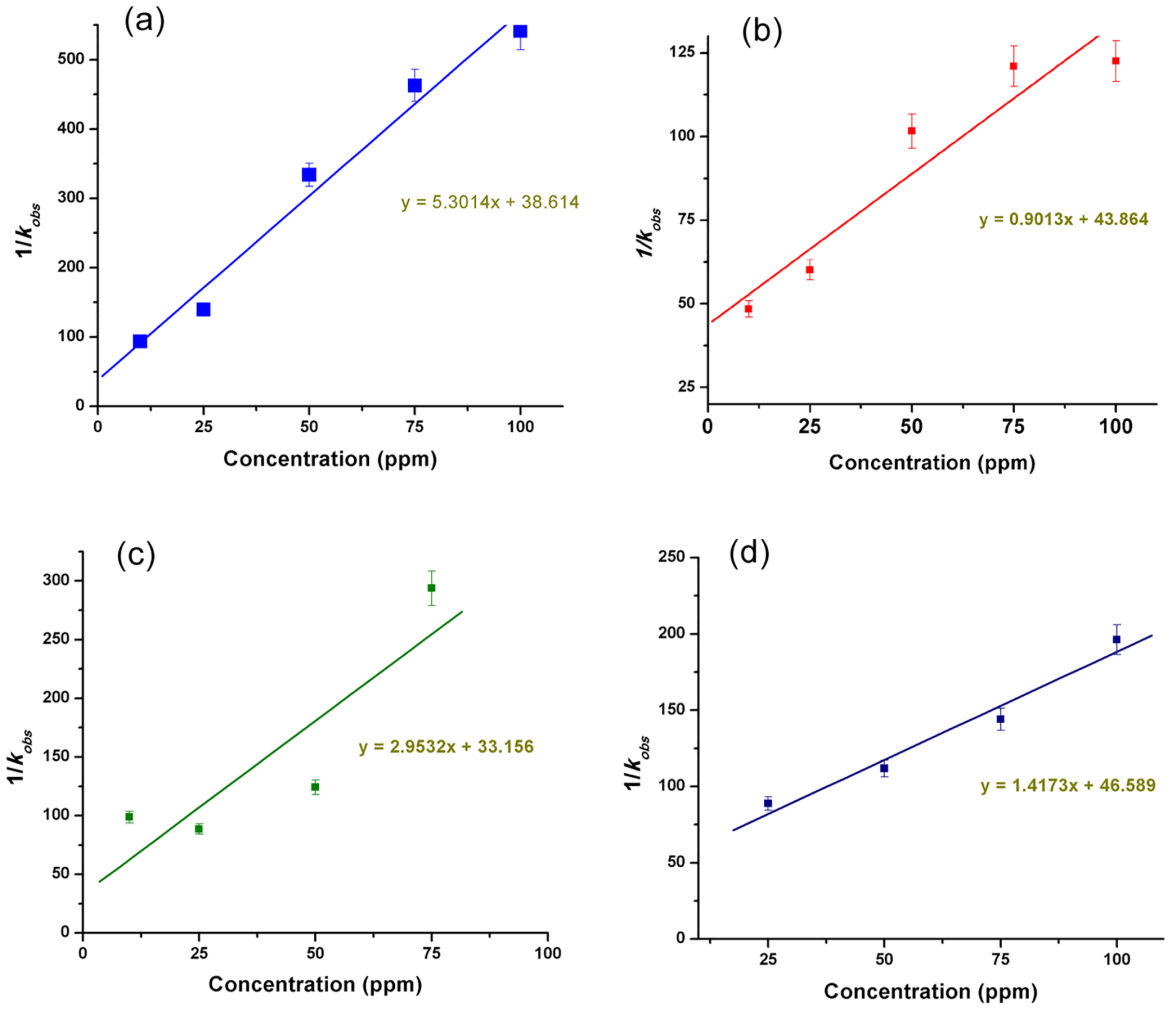
Plots representing the rate constants vs. initial concentrations of (a) benzene, (b) toluene, (c) ethylbenzene and (d) xylene.

The initial concentration of each monoaromatic hydrocarbon of BTEX was varied from 10 to 100 ppm and the values of k_obs_ was determined for each initial concentrations using a first order exponential fitting to their photocatalytic degradation curves (as shown in Fig 5). In Fig 6 we have plotted 1/ k_obs_ vs. initial concentration of each monoaromatic hydrocarbon of BTEX which shows a linear relationship between the initial concentration and 1/ k_obs_ confirming the L-H kinetic relationship [75,76]. The values of k_c_ and K_LH_ were then determined from the slope and Y-axis intersection of the linearly fitted line respectively, that are summarized in Table 1. Amongst the individual BTEX components, toluene shown maximum kc value of 1.109 mg.L^−1^.min^−1^ with lowest K_LH_ (0.0205 L.mg^−1^) indicating maximum adsorption on the ZnO nanorod surface, followed by xylene, ethylbenzene and finally benzene. These observations clearly conform to our results on the photocatalytic degradation efficiency of the monoaromatic hydrocarbons of BTEX as shown in Fig 5.

**Table 1:** Langmuir-Hinshelwood adsorption equilibrium constant (KLH) and rate constant for surface reaction (k_c_) values of BTEX obtained when individual BTEX molecules were photocatalytically treated using supported zinc oxide nanorods under visible light irradiation.

Chemical	k_c_	K_LH_
	(mg.L^−1^.min^−1^)	(L.mg^−1^)
Benzene	0.189	0.1373
Toluene	1.109	0.0205
Ethylbenzene	0.339	0.0891
Xylene	0.706	0.0304

**Fig 7:**
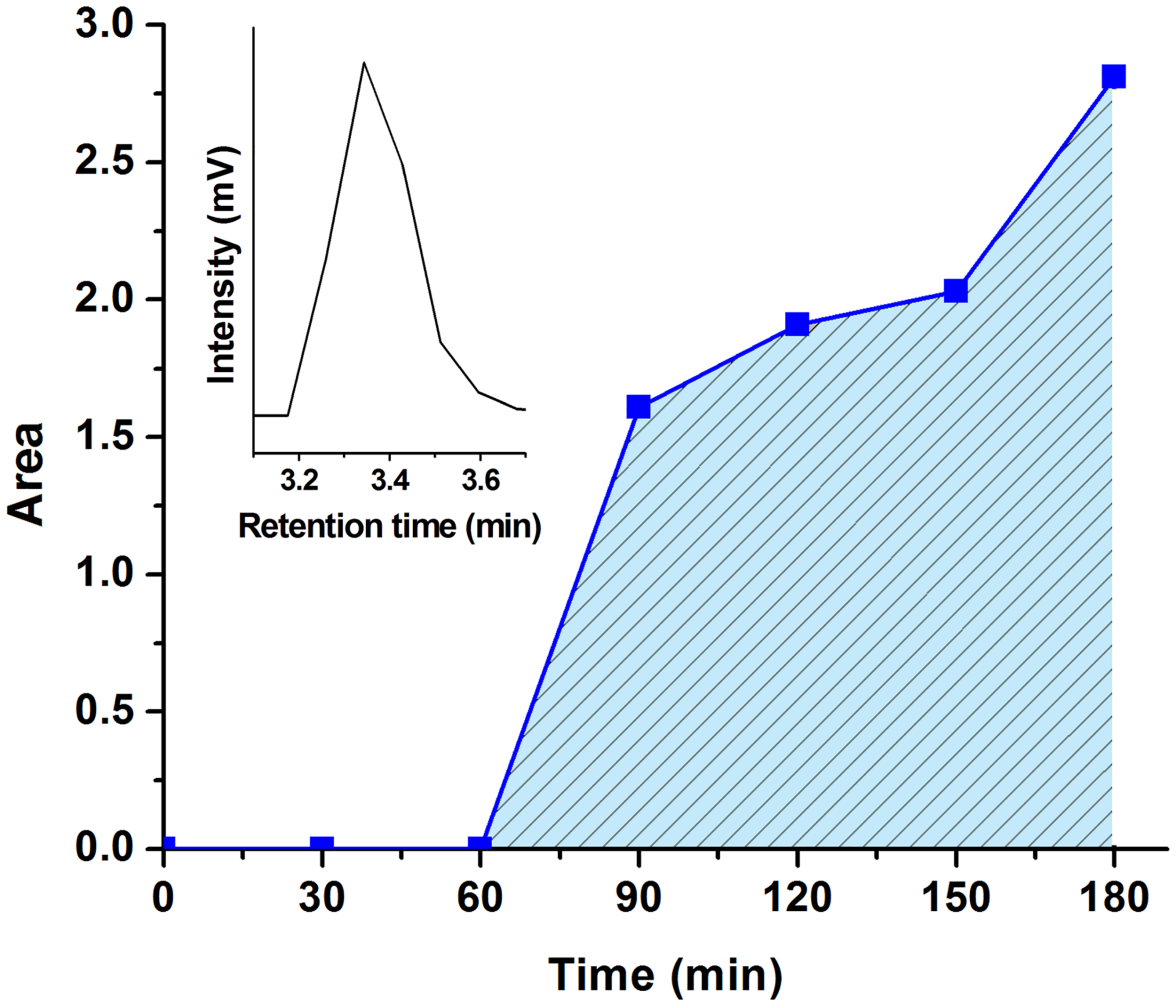
Evolution of carbon dioxide over time during BTEX photocatalytic degradation. Evolution of carbon dioxide over time degradation of 25 ppm BTEX mixture in DI water in the presence of ZnO nanorods under the simulated solar light irradiation as determined by using GC fitted with thermal conductivity detector (TCD). Inset shows the CO_2_ chromatogram detected at retention time 3.34 minutes.

**Fig 8:**
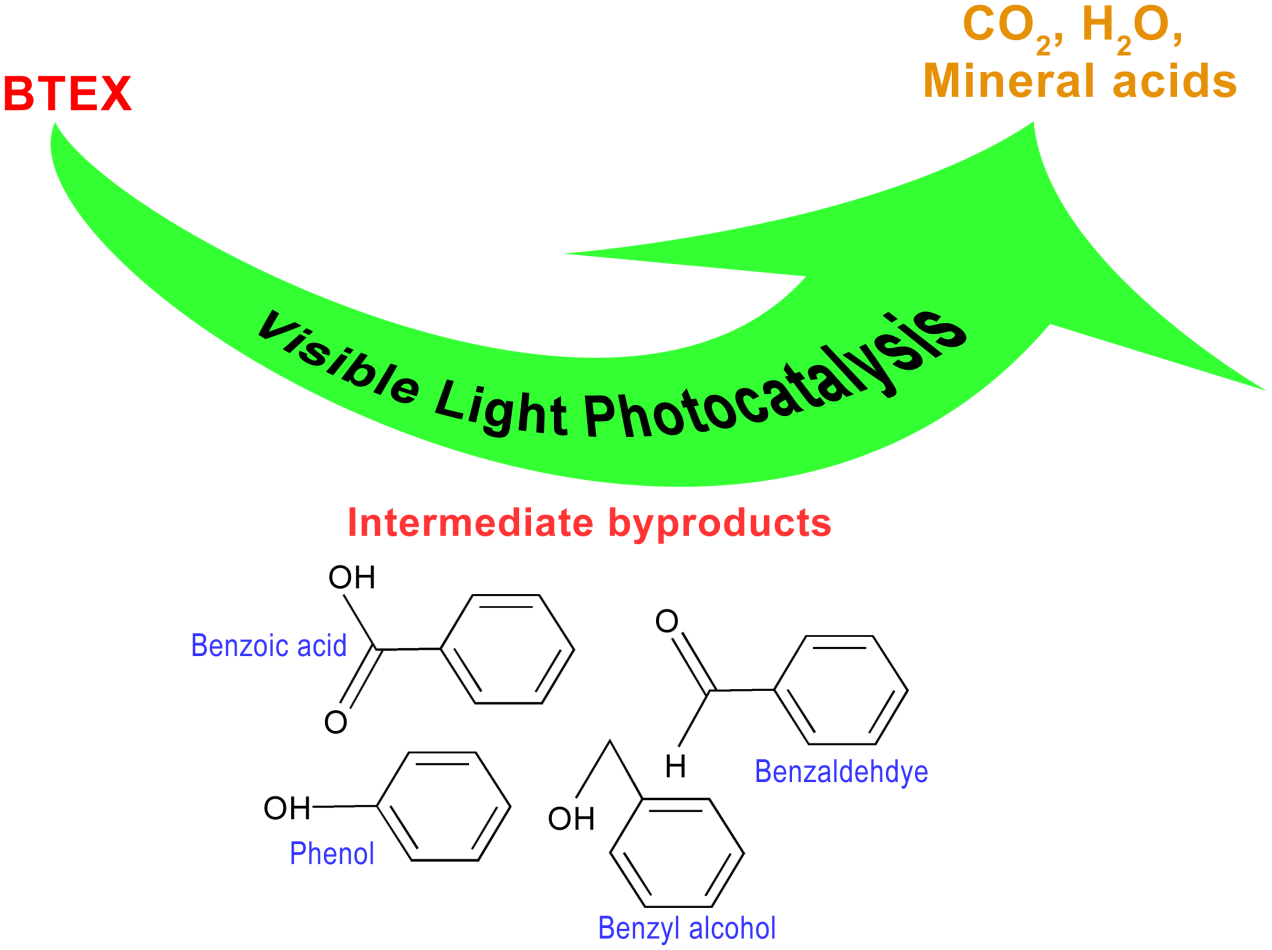
Schematic representation of visible light photocatalytic degradation of BTEX. Visible light photocatalytic degradation of BTEX in aqueous medium in the presence of ZnO nanorods as supported photocatalyst. Upon photo excitation, BTEX molecules are photocatalytically degraded by the ZnO nanorods resulting in various types of intermediate byproduct, which upon successive photo-oxidation produces CO_2_, H_2_O and mineral acids leading to the complete mineralization of BTEX.

The complete mineralization process of BTEX was investigated using a 25 ppm BTEX mixture solution in DI water to better understand the mineralization of BTEX. Using the HPLC technique, benzyl alcohol, benzaldehyde, phenol and benzoic acid were detected as intermediate byproducts that formed during the photocatalytic degradation of BTEX (Supporting information S2 Fig). Detailed investigation of the formation of intermediates and visible light photocatalytic degradation pathway of BTEX is currently on going. Similar intermediate byproduct formation as a result of photocatalytic degradation of BTEX was also reported by several other researchers [56,77–80]. Production of CO_2_ as a result of successive photo-oxidation of BTEX and the intermediate byproducts was detected after 1 hour of continuous photocatalytic degradation of BTEX with ZnO nanorods as shown in Fig 7, where CO_2_ content was observed to increase continuously after 1 hour indicating complete mineralization of BTEX mainly into CO_2_ and water. Based on these observations, the VLP degradation of BTEX with ZnO nanorods as a photocatalyst is schematically represented in Fig 8.

## Conclusions

Microwave assisted hydrothermal process was used successfully to grow ZnO nanorods on glass support and used as supported visible light photocatalyst to degrade BTEX in aqueous medium. Presence of native point defects, mainly zinc interstitial and oxygen vacancy states extend the absorption edge of the ZnO nanorods into the visible region. As a result, efficient visible light photocatalytic (VLP) degradation of BTEX was observed in the presence of ZnO nanorods, resulting in almost 90% reduction in toluene and xylene concentrations as well as ~80% reduction in ethylbenzene and ~65% reduction in benzene within 3 hours. Langmuir-Hinshelwood kinetic model fits well with the experimental results showing a maximum reaction and adsorption/desorption equilibrium constants for toluene with the ZnO nanorod surface, followed by xylene, ethylbenzene and benzene, respectively. Further investigation of photocatalytic degradation of BTEX by ZnO nanorods as visible light photocatalyst exhibited formation of benzyl alcohol, benzaldehyde, phenol and benzoic acid as intermediate byproducts. CO_2_ evolution as a result of successive photo-oxidation of BTEX and its intermediate byproducts was also evaluated which was found to increase over time indicating the final mineralization of the BTEX molecules into benign products.

## Supporting information

S1 Fig GC/MS chromatogram of BTEX. (DOCX)

S2 Fig HPLC chromatogram of BTEX intermediate products. (DOCX)
