# Supplementary material for: Efficient visible light photocatalysis of benzene, toluene, ethylbenzene and xylene (BTEX) in aqueous solutions using supported zinc oxide nanorods
Source: PLoS One. 2017 Dec 20;12(12):e0189276. doi: 10.1371/journal.pone.0189276 (PMC5738043; doi:10.1371/journal.pone.0189276)
Supplement: S1 Fig — (DOCX) [file pone.0189276.s001.docx]

**Supporting Information**

**Efficient visible light photocatalysis of benzene, toluene, ethylbenzene and xylene (BTEX) in aqueous solutions using supported zinc oxide nanorods**

**Jamal Al-Sabahi ^1,2^ , Tanujjal Bora ^2^ , Mohammed Al-Abri ^1,2,*^ and Joydeep Dutta ^3,*^**

^1^ Department of Petroleum and Chemical Engineering, College of Engineering, Sultan Qaboos University, PO Box 33, PC 123, Al-Khoudh, Oman

^2^ Chair in Nanotechnology for Water Desalination, Water Research Center, Sultan Qaboos University, PO Box 17, PC 123, Al-Khoudh, Oman

^3^ Functional Materials Division, Materials and Nanophysics, ICT School, KTH Royal Institute of Technology, Isafjordsgatan 22, Kista Stockholm SE-164-40, Sweden

* Corresponding author: alabri@squ.edu.om (+968-2454-3794); joydeep@kth.se (+46-73-765 21 86)

**S1 Fig.** **GC/MS chromatogram of BTEX in aqueous solution**

GC/MS chromatogram of BTEX aqueous solution (25 ppm) representing the peaks of benzene, toluene, ethylbenzene and xylene that were detected at retention time 3.58, 5.33, 7.92 and 10.17 minutes respectively.
