# Supplementary material for: Efficient visible light photocatalysis of benzene, toluene, ethylbenzene and xylene (BTEX) in aqueous solutions using supported zinc oxide nanorods
Source: PLoS One. 2017 Dec 20;12(12):e0189276. doi: 10.1371/journal.pone.0189276 (PMC5738043; doi:10.1371/journal.pone.0189276)
Supplement: S2 Fig — (DOCX) [file pone.0189276.s002.docx]

**Efficient visible light photocatalysis of benzene, toluene, ethylbenzene and xylene (BTEX) in aqueous solutions using supported zinc oxide nanorods**

**Jamal Al-Sabahi ^1,2^ , Tanujjal Bora ^2^ , Mohammed Al-Abri ^1,2,*^ and Joydeep Dutta ^3,*^**

^1^ Department of Petroleum and Chemical Engineering, College of Engineering, Sultan Qaboos University, PO Box 33, PC 123, Al-Khoudh, Oman

^2^ Chair in Nanotechnology for Water Desalination, Water Research Center, Sultan Qaboos University, PO Box 17, PC 123, Al-Khoudh, Oman

^3^ Functional Materials Division, Materials and Nanophysics, ICT School, KTH Royal Institute of Technology, Isafjordsgatan 22, Kista Stockholm SE-164-40, Sweden

* Corresponding author: alabri@squ.edu.om (+968-2454-3794); joydeep@kth.se (+46-73-765 21 86)


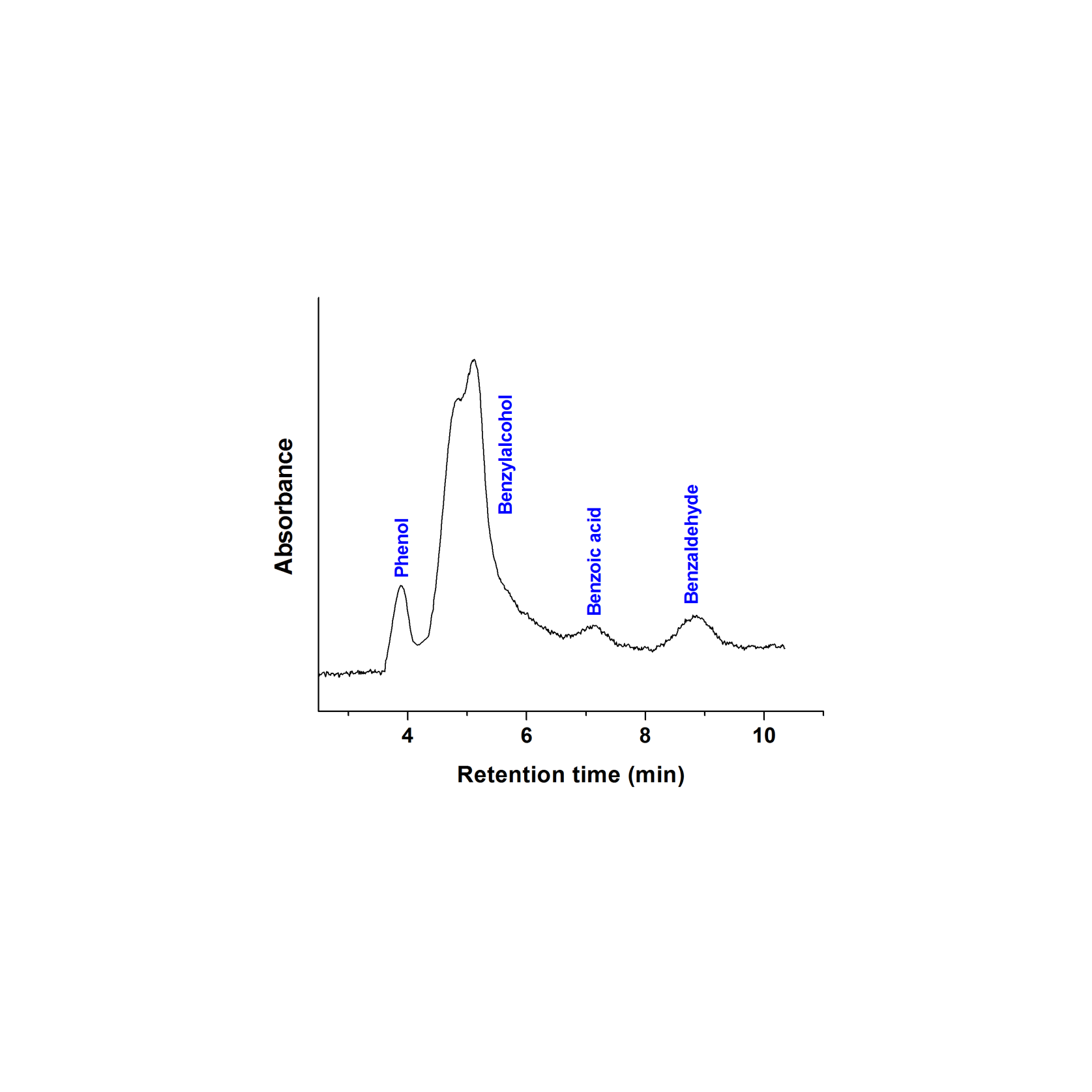


**S2 Fig.** **HPLC chromatogram of intermediate byproducts**

HPLC chromatogram representing the intermediate byproducts detected as a result of the photocatalytic degradation of BTEX under visible light irradiation in the presence of ZnO nanorods as photocatalyst. The chromatogram was recorded after 90 minutes of the photocatalytic reaction, where peaks of phenol, benzyl alcohol, benzoic acid and benzaldehyde were found at retention time 3.88, 5.12, 7.11 and 8.80 minutes respectively. The detailed analysis of intermediate byproducts produced during the photocatalytic degradation of aqueous BTEX solution is currently ongoing and will be reported elsewhere.
